# A Novel Coupling Mechanism for CSRRs as Near-Field Dielectric Sensors

**DOI:** 10.3390/s22093313

**Published:** 2022-04-26

**Authors:** Ali M. Albishi

**Affiliations:** Electrical Engineering Department, King Saud University, Riyadh 11421, Saudi Arabia; aalbishi@ksu.edu.sa

**Keywords:** inductive coupling, capacitive coupling, microwave sensors, near-field sensors, electrically small resonators, complementary split-ring resonators, sensitivity enhancement

## Abstract

This work proposes a novel coupling mechanism for a complementary split-ring resonator as a planar near-field microwave sensor for dielectric materials. The resonator is etched into the ground plane of a microstrip line. This mechanism is based on the inductive coupling synthesized by utilizing a via that connects the power plane of the microstrip line to the central island of the resonator. The proposed coupling makes the coupling capacitance between the transmission line and the resonator relatively small and insignificant compared to the capacitance of the resonator, making it more sensitive to changes in the dielectric constant of the materials under test. In addition, the coupling is no longer dependent solely on the capacitive coupling, which significantly reduces the coupling degradation caused by loading the resonator with dielectric materials, so the inductive coupling plays an important role in the proposed design. Therefore, the proposed coupling mechanism improves the sensitivity and enhances the coupling between the transmission line and the resonator. The sensor is evaluated for sensitivity, normalized resonance shift, and coupling factor using a full-wave numerical simulation. The sensitivity of the proposed sensor is 12% and 5.6% when detecting dielectric constants of 2 and 10, respectively. Compared to recent studies, the sensitivity improvement when detecting similar permittivity is 20% (1.32 times) and 9.8% (1.1 times). For verification, the proposed sensor is manufactured using PCB technology and is used to detect the presence of two dielectric laminates.

## 1. Introduction

There has been great interest in developing microwave resonators based sensors for a variety of applications, such as in the biomedical and food industries, and for microfluidic applications, the surface crack detection of solid materials, and material characterizations, to name but a few [1,2,3,4,5,6,7,8,9,10,11]. For example, many applications are based on the fluidic and microfluidic analyses that are part of some technologies, such as the lab-on-a-chip technology [12]. In analysis-based systems, it can be argued that it is difficult to find a system that does not utilize sensors, which are essential to interact with materials under test (MUTs). Therefore, sensors can be regarded as the heart of such systems, requiring inexpensive, sensitive, selective, responsive, and unlabeled sensors [8]. Among other resonators, microwave sensors based on electrically small resonators, such as split-ring resonators (SRRs) and complementary split-ring resonators (CSRRs), have shown great potential to be utilized to design such systems.

Planar microwave resonators based on electrically small resonators have become attractive in recent years for designing different detection modalities. The resonators can be modeled approximately using lumped-circuit models that can be used to analyze resonators in terms of their inductive, capacitive, and resistive elements. When coupled to two-port transmission lines, e.g., microstrip lines, the resonators exhibit stop-band responses and can be utilized to design stop-band filters for filter applications [13,14,15]. At the resonance frequency, the transmission coefficient $|S_{21}|$ becomes minimum, and circuit models near this frequency can be extracted. Here, the resonant frequency can be expressed as a function of the inductance and the capacitance. The resonators’ capacitances can be loaded with the MUTs and utilized as near-field sensors. For planar resonators, the MUTs can be modeled as a capacitance added in parallel with the resonators’ capacitances, thus shifting down the resonant frequency to a lower frequency.

Electrically small resonators include split-ring resonators (SRRs) and complementary split-ring resonators (CSRRs). Whether the first proposed electrically small resonator is a split-ring resonator (SRR) is controversial or not, and it can be traced back to 1952 [16]. Then, in 1999, SRR was introduced by Pendry to design metamaterials [17]. SRR and its complement (CSRR) have been attractive and have been utilized in many state-of-the-art technologies, such as for glucose blood detection [11,18], breast imaging systems [19], mutual coupling suppression [20,21], filters [13,14,15], and antennas [22,23,24,25,26]. At the resonance frequency of the resonators, the electromagnetic energy is concentrated in a small volume [17], making the resonators sensitive to changes in the nearby environment. Therefore, the resonators have been adopted for designing sensors for different applications, such as biomolecular applications [7,27,28,29], the crack detection of solid materials [4,5,30], microfluidic applications [6,31,32], material detection [33,34,35], concentration measurements [36], and dielectric and liquid characterizations [27,37,38,39,40,41,42,43,44].

For microfluidic technologies that require miniaturized detection areas, SRRs are more suitable than CSRRs for the sensor design. In fact, SRRs have been adopted to design microwave heating and detection systems [45,46,47,48]. However, the resonators suffer from limited sensitivity for many reasons, such as the electric field concentration in the substrate limiting the field’s ability to interact with the MUTs [35,49] and the small detection areas. Thus, many proposed techniques have been introduced to overcome such limitations [35,43,44,49,50]. The sensor proposed in reference [50] shows relatively high sensitivity, but the structure is no longer planar.

CSRRs, on the other hand, are highly sensitive to dielectric changes [37] and have been shown to have high coupling between transmission lines and the resonators; yet, the sensors still have some limitations detecting higher dielectric materials. The coupling depends fundamentally on the capacitance-per-unit length of the transmission lines (TLs), which can be affected by loading the resonators with high dielectric materials since the electric field will be eventually be concentrated out of the substrates (e.g., in the MUTs). In addition, it has been shown in reference [51] that the sensitivity of defected ground resonators such as CSRRs can be improved by eliminating the coupling capacitance. However, the coupling capacitance is essential for coupling. Therefore, it can be seen that the resonators as stop-band-based sensors have inherent limitations associated with the coupling capacitance. Furthermore, the sensing system requires $|S_{21}|$ measurements. This needs at least two-port VNAs, making it difficult to utilize the resonators to design fully integrated systems. Thus, further investigation is needed to find a solution that achieves the following:

1. The reduction of the need for two-port measurements, since it will be relatively easier and cheaper to design an apparatus that is used to measure the return loss or the reflection coefficient $|S_{11}|$ instead of $|S_{21}|$. Thus, one-port scalar network analyzers are only needed;
2. The complete or partial elimination of the need for the coupling capacitance that limits the sensitivity of the resonator-based systems as well as the coupling, especially for a high dielectric load;
3. The utilization of transmission lines (e.g., microstrip line technology) to excite the resonators, as such an excitation mechanism has its own advantages, since the TL can be regarded as a quasi-TEM in which the MUTs are loading the discontinuities (the resonators), not the whole TLs. In addition, the one-port TLs are easy to integrate with the current circuit technologies.

In this paper, we propose for the first time a new coupling mechanism based on inductive coupling to excite CSRR. Note that, the inductive coupling is a common technique as a coupling mechanism to improve devices’ performances. However, it has not been utilized to excite CSRRs as near-field sensors. The resonator is coupled to the TL through an inductive element, reducing the need for capacitive coupling. Therefore, our proposed sensor has the following advantages. The TL-to-resonator coupling is enhanced, even with higher dielectric materials, reducing the need for the coupling capacitance. The extracted lumped elements based on a proposed circuit model show that the coupling capacitance becomes relatively small and insignificant compared to the resonator’s capacitance. In fact, the analysis has shown that the coupling is improved when the resonator is loaded with MUTs. In addition, the system exhibits a band-pass response (minimum reflection) that only requires the measurement of the return loss or the reflection coefficient. The sensitivity of the proposed sensing system is enhanced even with the comparison to a recent paper [40]. Although the proposed sensor in reference [40] shows sensitivity enhancement, the proposed sensor in reference [40] is only based on an optimization routine. In our work, we provide a complete numerical analysis and theory for realizing and synthesizing the proposed sensor. The proposed sensor was evaluated using a 3D numerical simulation and experimentation. The sensor is fabricated using PCB technology and tested using a VNA. Two samples of dielectric materials were selected to validate the sensor’s improved sensitivity as well as the enhanced coupling as a dielectric sensor. Therefore, we emphasize that this paper is intended to validate the proposed theory for coupling and sensing enhancement rather than targeting a certain application.

## 2. Theory of Coupling and Sensitivity Enhancement

**Figure 1:**
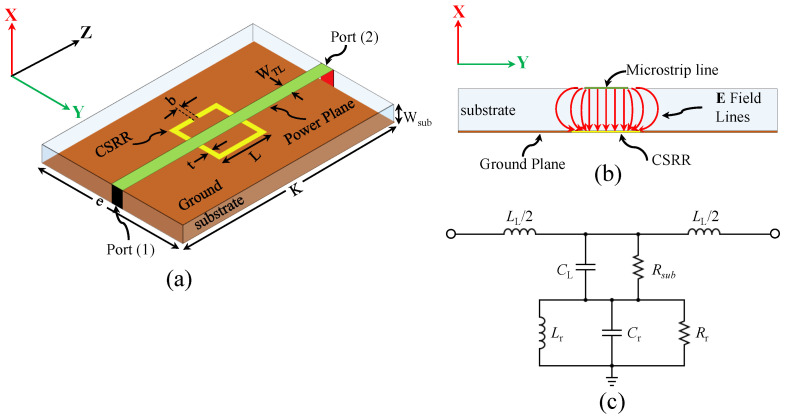
(**a**) The schematic of the original CSRR excited using two-port microstrip line, $W_{\mathrm{sub}}$ = 0.762 mm, $W_{\mathrm{TL}}$ = 1.629 mm, e = 40 mm, K = 100 mm, L = 7.5 mm, t = 0.2 mm, and b = 0.2 mm. (**b**) The cross-section of the CSRR sensor with the expected electric field lines of the quasi-TEM TL. (**c**) The circuit model of the CSRR, where $L_{L}$ is the inductance-per-unit length of the TL, $C_{L}$ is the capacitance-per-unit length of the TL, $R_{sub}$ is losses in the substrate, $C_{r}$ is the resonator’s capacitance, $R_{r}$ is the resonator’s resistance, and $L_{r}$ is the resonator’s inductance.

As near-field sensors, defected-ground resonators such as CSRRs can be excited using microstrip lines, where the electric field lines are perpendicular to the surface of the resonators. Figure 1a shows a CSRR-based sensor where the resonator is etched out in the ground plan of a two-port 50 Ω microstrip line, whereas Figure 1b shows the cross-section of the sensor with the expected electric field lines of the quasi-TEM TL.

CSRR can be approximately modeled using lumped elements around the resonance frequency [52]. The normal electric field component, shown in Figure 1b, can be modeled as a parallel capacitance ($C_{L}$) between the power plane and ground plane, whereas the magnetic field of the TL is modeled as an inductance $L_{L}$. The dielectric loss of the substrate can be modeled as a resistance ($R_{sub}$). The electric field will create a potential difference between the central island of the resonator and the ground plane. The potential difference, modeled as a capacitance of the resonator ($C_{r}$), will create a circulating surface current, modeled as an inductance ($L_{r}$) in both sides of the resonator (the substrates and the free space), whereas the effective losses in the resonator can be modeled as a resistance ($R_{r}$). Based on the circuit model, the system will exhibit a minimum transmission of $|S_{21}|$ at a certain frequency corresponding to the dimensions of the resonator, and the resonance frequency is given by references [53,54]

(1) $$f_{z0}=\frac{1}{2\pi \sqrt{L_{r}(C_{r}+C_{L}})}$$

When the resonator encounters a change in the potential difference by loading it with MUTs, the effective capacitance will be increased since the MUTs can be seen as a capacitance ($C_{MUT}$) added in parallel to $C_{r}$, thereby lowering the resonance frequency. Consequently, the resonator can be used to design near-field sensors. It has been reported that the sensitivity of CSRRs depends on $C_{L}$ [51], so minimizing the dependency of the sensor on the $C_{L}$ (e.g., $C_{L}=0$) would improve the sensitivity. The sensitivity of the CSRRs is derived as in reference [51],

(2) $$S=\frac{f_{z0}}{\Delta \epsilon }\left(\frac{1}{\sqrt{\frac{\left(\alpha -1\right)C_{r}}{C_{L}+C_{r}}}+1}-1\right)$$

where α is a real number associated with the increase in $C_{r}$ when loaded with the MUTs (e.g., $C_{r(new)}$∝α$C_{r(old)}$). Therefore, the maximum sensitivity of the CSRRs can be obtained by letting $C_{L}$ be zero [51]. However, $C_{L}$ is an important parameter for exciting the resonator and cannot be eliminated. In addition, as the permittivity of the MUT increases, the electric field eventually concentrates more inside the MUT and $C_{L}$ consequently decreases. Again, the response of the sensor ($|S_{21}|$) will eventually vanish. The effects of the high-dielectric materials on the coupling capacitance can be evaluated by calculating the coupling factor. The traditional CSRR loading a microstrip line can be categorized as a band-gap-based resonator in which the coupling factor can be obtained as in reference [55]

(3) $$\kappa =\frac{1-|S_{21(f_{z0})}|}{|S_{21(f_{z0})}|}$$

It has been reported that the coupling factor (κ) of the traditional CSRRs excited by the capacitive coupling of the TLs decreases significantly as the dielectric constant of the MUTs increases [56].

**Figure 2:**
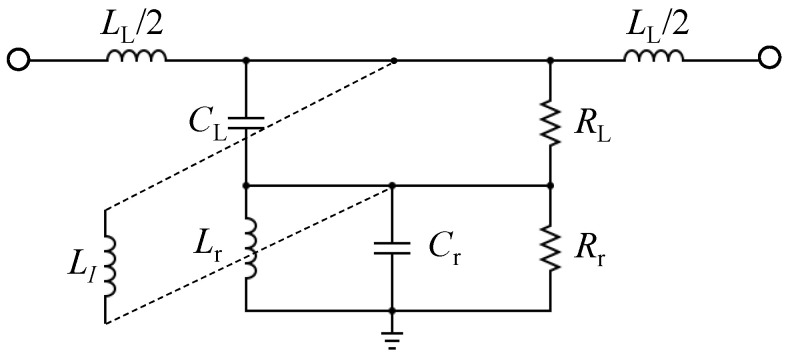
The proposed circuit model of the CSRR with the inserted via.

Consequently, it can be concluded that reducing the dependency of the CSRRs-based sensors on the capacitive coupling of the TLs will have the effect of improving simultaneously the sensitivity and the coupling. This can be investigated by considering other types of coupling, such as inductive coupling. By reconsidering the circuit model of the CSRR shown in Figure 1c, one can insert an inductor ($L_{I}$) that is parallel to $C_{L}$, as shown in Figure 2. Based on the modified circuit model, it can be predicted that the system will exhibit a band-pass response based on the values of the shunt circuit tank.

**Figure 3:**
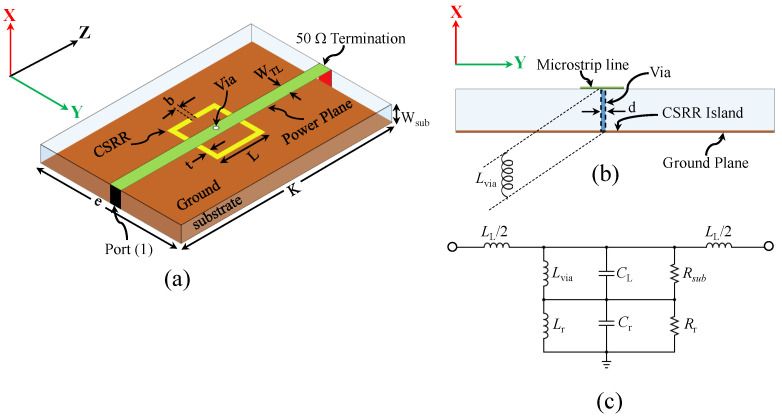
(**a**) The schematic of the proposed CSRR excited using one-port microstrip line where the power plane is connected to the ground plane using a via, d = 0.2 mm, $W_{\mathrm{sub}}$ = 0.762 mm, $W_{\mathrm{TL}}$ = 1.629 mm, e = 40 mm, K = 100 mm, L = 7.5 mm, t = 0.2 mm, and b = 0.2 mm. (**b**) The cross-section of the CSRR with the via with where the inserted via can be seen as a parallel inductance. (**c**) The circuit model of the CSRR, where $L_{via}$ is the inductance of the inserted via, $L_{L}$ is the inductance-per-unit length of the TL, $C_{L}$ is the capacitance-per-unit length of the TL, $R_{sub}$ is losses in the substrate, $C_{r}$ is the resonator’s capacitance, $R_{r}$ is the resonator’s resistance, and $L_{r}$ is the resonator’s inductance.

The inserted inductance ($L_{I}$) can be synthesized using a via, where the power plane of a microstrip line is connected to the center island of a CSRR. Figure 3a shows the proposed sensor, whereas Figure 3b,c show the side view of the sensor and the expected circuit model, respectively. Note that, in the proposed sensor, the inserted inductance ($L_{I}$) is denoted as $L_{via}$ to emphasize its importance. Thus, based on the investigation in reference [51] and the proposed new coupling mechanism, it is expected that the sensitivity and the coupling factor of the CSRR will be enhanced and can be utilized as a dielectric sensor.

## 3. Sensor Design, Numerical Analysis, and Discussion

Since the proposed sensor will be evaluated using a 50 Ω VNA, a 50 Ω microstrip line will be designed to excite the resonator. There is no intrinsic reason for choosing the CSRR’s length except for the suitability to measure the response within the frequency range of our VNA and to make the sensor work at the lower microwave regime, which can be relatively associated with the low-cost fabrication and being relatively easy for integration with other systems. In addition, we can evaluate and quantify the sensitivity enhancement in comparison to our work in reference [52]. Nevertheless, the sensitivity of the sensor has to be normalized if one wants to make a fair comparison with other published microwave sensors. Therefore, the following normalized sensitivity [50] and the normalized-shift resonance frequency [40] as a figure of merit will be used for the evaluation,

(4) $$S=\frac{\Delta f}{f_{0}\left(\epsilon_{r}-1\right)}\times 100$$

(5) $$\mathrm{Norm}\Delta f=\frac{\Delta f}{f_{0}}\times 100$$

where $f_{0}$ is the operating resonance frequency, Δf is the relative resonance frequency shift compared to the reference case (e.g., the air), and $\epsilon_{r}-1$ is the relative variation in the permittivity.

For the evaluation, two CSRRs-based sensors, with and without a via, were designed and investigated utilizing the full-wave simulation (ANSYS-HFSS). Utilizing a low-loss substrate from Rogers materials (the Rogers RO4350) with the dielectric constant of 3.66 (the effective dielectric constant ≈ 2.85) and the thickness of $W_{\mathrm{TL}}$ = 0.762 mm, 50 Ω microstrip lines with a width of $W_{\mathrm{sub}}$ = 1.629 mm were synthesized. Table 1 shows the design specification for the CSRR sensors with and without the via. With the design specification of the CSRR (no via), the sensing system exhibits a minimum transmission coefficient ($|S_{21}|$) at the frequency (the resonance frequency) of $f_{0}$ = 3.22 GHz. By considering the physical length of the resonator in the direction of the propagation (L = 7.5 mm), the effective dielectric constant is $\epsilon_{eff}$ = 2.85, the guided wavelength $\lambda_{g}=\lambda_{air}/\sqrt{\epsilon_{eff}}$ = 55 mm, and the relative length of the resonator is $\lambda_{g}/7.3$. For the CSRR with the via, the sensing system exhibits a minimum reflection coefficient ($|S_{11}|$) at the frequency (the resonance frequency) of $f_{0}$ = 5.092 GHz, where the relative length is $\lambda_{g}/4.6$.

**Figure 4:**
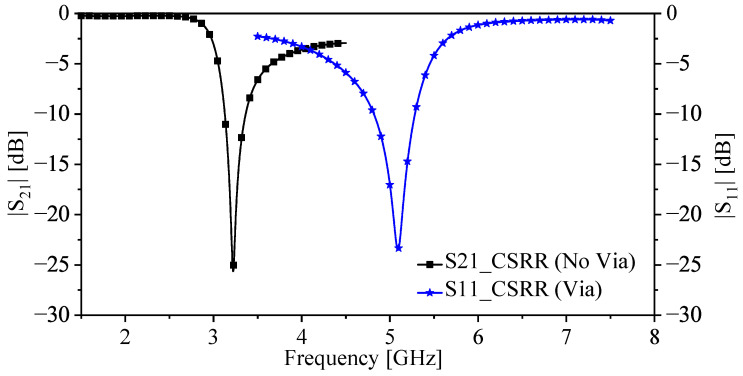
The responses of the original CSRR ($|S_{21}|$ [dB]) and the proposed sensor ($|S_{11}|$ [dB]), where the resonator’s length L = 7.5 mm.

For both designs, the systems are analyzed numerically using HFSS with 50 Ω ports (port one and port two). Thus, the response of the systems is characterized using $|S_{21}|$ and $|S_{11}|$. Both responses are utilized to extract the circuit models shown in Figure 1c and Figure 3c for the systems without and with the via, respectively. Now, since the system without the via exhibits a stop-band response, where the resonance frequency can be observed at the minimum $|S_{21}|$, the system has been utilized as a near-field sensor in which the sensing mechanism is based on observing the resonance frequency shift. On the other hand, the system with the via (the proposed system) exhibits a band-pass response, where the resonance frequency can be observed at the minimum $|S_{11}|$ and the system can be utilized as a near-field sensor in which the sensing mechanism is based on observing the resonance frequency shift. Thus, as a near-field sensor, it is only needed to measure $|S_{11}|$ either by terminating the second port by a 50 Ω load or connecting the second port of the system to the second port of a 50 Ω VNA. Figure 4 shows the response of the CSRR sensors without ($|S_{21}|$) and with the via ($|S_{11}|$). By comparing the relative length of the two resonators, where the physical length is fixed (L = 7.5 mm), the CSRR with the via is seen to be electrically larger, making its sensing area larger and, hence, the sensitivity enhancement is expected.

Although the relative length of the CSRR is increased by a factor of 58.7%, the resonator can still be dealt with as an electrically small resonator. The approximation of being considered as an electrically small resonator will help to extract an equivalent-circuit model near the resonance frequency. Of course, the circuit model based on lumped elements can be validated by comparing the responses of the numerical simulation and the lumped-elements-based model. The circuit model will help to investigate the effect of the via, where the coupling capacitor ($C_{L}$) will be evaluated before and after inserting the via. The process of extracting the circuit models is rather straightforward. The responses of the systems (($|S_{21}|$) and ($|S_{11}|$)) with and without the via were obtained first by the numerical simulation by Ansys-HFSS and then imported to the circuit model simulation by Keysight-ADS. By utilizing the optimization toolbox provided in the simulator, the circuit parameters, shown in Figure 1c and Figure 3c, were extracted and presented in Table 2.

**Figure 5:**
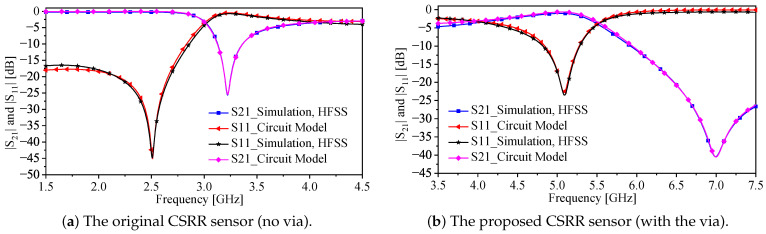
(**a**) The response ($|S_{11}|$ [dB] and $|S_{21}|$ [dB]) of the original (**b**) and the proposed CSRR (L = 7.5 mm) extracted using the simulation and the equivalent-circuit model that is presented in Figure 1c and Figure 3c, respectively.

**Figure 6:**
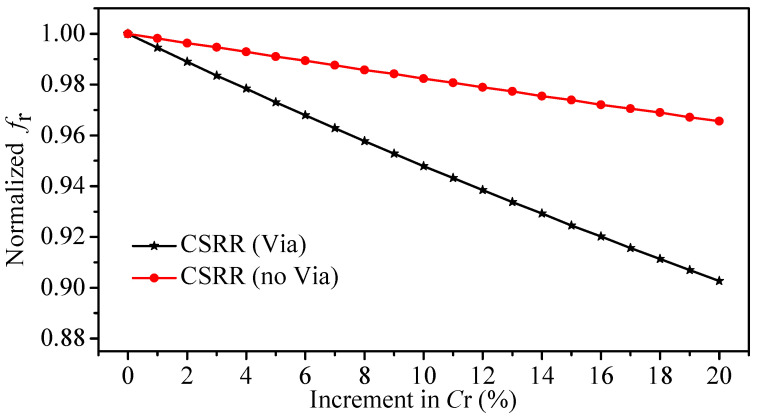
The normalized resonance frequency extracted from the circuit models versus the increment in $C_{r}$ (in percentage) for the original CSRR and the proposed CSRR.

Figure 5a,b show the responses of the CSRRs sensors without and with the via, respectively. It is worth mentioning that the lumped-elements model is only acceptable close to the resonance frequency. The MUTs can be seen as parallel capacitance to the $C_{r}$ for both resonators where the circuit model can be adopted to evaluate the sensitivity of the resonance frequency to small changes in the $C_{r}$ [57]. Since we are only interested in small and relative changes, the increment in the value of $C_{r}$ was expressed in percentage (0% to 20% incremental). In addition, the resonance frequencies were normalized with respect to the reference case (the air). Figure 6 shows the resonance frequency (normalized) versus the increment in the $C_{r}$ in percentage. By comparing the slope of the two curves in Figure 6 for the CSRR with (slope = −4.86 × 10${}^{-3}$) and without (slope = −1.72 × 10${}^{-3}$) the via (note that, we assumed that the two curves are linear), the enhancement in the sensitivity of $f_{r}$ to a small change in $C_{r}$ is seen to be 182.55%.

**Figure 7:**
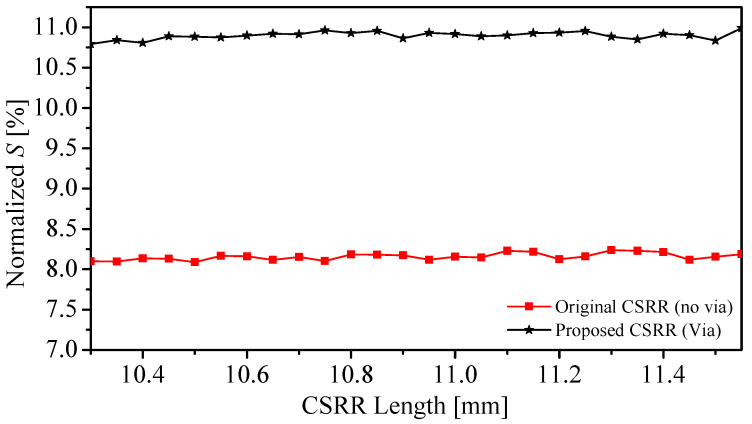
The normalized sensitivity versus the resonator’s length, which is varied from 10.3 to 11.55 mm for the original CSRR sensor and the proposed CSRR sensor.

Furthermore, the sensitivity of the two sensors to detect the changes in the dielectric MUTs versus the length of the resonators was investigated. The dielectric constant of a slab, with thickness = 3 mm and width = length = 22.5 mm, was varied from one (the reference case) to two, with a step of one so that the relative variation in the permittivity $\epsilon_{r}-1$ is one ($\Delta \epsilon_{r}$ = 1). In other words, the normalized *S* becomes a function of the resonator length at $\Delta \epsilon_{r}$ = 1. Note that we utilized the proposed sensor with a similar configuration, but with a different resonator’s length. Thus, at different lengths, we have effectively different resonators with their own electromagnetic field distributions in which each resonator will have a different sensitivity, so we should not expect that they will follow a defined pattern. In fact, this is one of the advantages of utilizing the normalized sensitivity since it reveals the difference in the sensitivity of different resonator lengths. Using the resonance frequency or the shift in the resonance frequency without normalization might give a misleading indication that increasing the length will degrade the sensitivity, which is not the case when we use the normalized sensitivity or normalized resonance frequency shift, as evident from Figure 7. The choice of the thickness of the slab is based on the fact that the electromagnetic fields of these types of resonators are highly concentrated in the proximity of the sensors [58]. By using (4), two curves, shown in Figure 7 (CSRR-No Via) and (CSRR-Via), were produced that can be utilized in the sensor design, such as a lookup table where the sensitivity versus the length can be specified by the designated applications as well as the frequency range.

Moreover, since the proposed sensor can be categorized as a band-pass-based resonator, the coupling factor can be obtained as in reference [55].

(6) $$\kappa =\frac{1-|S_{11(f_{z0})}|}{|S_{11(f_{z0})}|}$$

Thus, the coupling factor was evaluated using (3) and (6) for the sensors (case one: L = 7.5 mm and case two: L = 10.2 mm) with and without the via, respectively, when detecting the presence of a dielectric slab with different dielectric constants (2, 10, 19, 30, 69, and 80). For instance, for L = 7.5 mm, the coupling factor for the proposed sensor was 22.95 and 36.2 with the dielectric constants of 2 and 80, respectively, whereas in the case of the CSRR without the via, the coupling factor is 14.86 and 0.35 with the dielectric constants of 2 and 80, respectively. It is evident that the coupling factor of the proposed sensor is less sensitive to the loading MUTs. The results of the coupling factor are summarized in Table 3.

**Figure 8:**
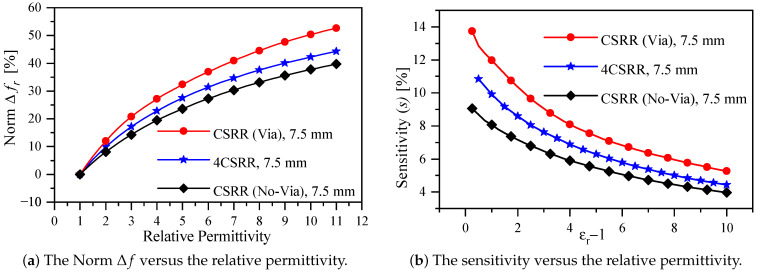
The sensor evaluation using (4) and (5), (**a**) the normalized resonance frequency shift versus the relative permittivity for three cases: the original CSRR sensor (no via), the 4CSRR sensor, and the proposed CSRR sensor (with via), whereas (**b**) the sensitivity versus the relative variation in the permittivity for three cases: the original CSRR sensor (no via), the 4CSRR sensor, and the proposed CSRR sensor (with via).

With the design specification shown in Table 1, the sensors with and without the via were utilized to detect the changes in the dielectric constant of a solid slab with a thickness = 3 mm and width = length = 22.5 mm. In the numerical simulation Ansys-HFSS, the dielectric constant of the slab was varied from 1 (the reference case) to 11 with the step of 0.25. This particular choice of the range will cover the two-dielectric slabs that will be used in the experimental validation. In addition, the results of the numerical simulation will be utilized to evaluate the improved sensitivity as well as the enhanced coupling with the comparison to the state-of-the-art electrically small resonators-based sensors. Figure 8a shows the normalized resonance shift versus the relative permittivity for three cases, namely the proposed sensor, the original CSRR (no via), and the 4CSRR (reported in [52]). The normalized resonance shifts of the proposed sensors when detecting the dielectric constants of 2 and 10 are 12% and 50.34%, respectively, whereas the sensitivities are 12% and 5.6%. In comparison to recent work [40,52], the enhancement in the normalized resonance shift is 20% (1.2 times) when detecting the dielectric constant of two, whereas the enhancement in the normalized resonance shift detecting the dielectric constant of 10 is 19.09% (1.19 times) and 9.43% (1.1 times) in comparison to references [40,52], respectively. It is noteworthy that the physical dimensions of the proposed sensor is much more compact relative to the excitation wavelength, 0.127$\lambda_{0}$ versus 0.2$\lambda_{0}$ in reference [40]. Furthermore, by using (4), the sensitivity versus the permittivity variation of the CSRR-based sensor with and without via can be calculated to evaluate the sensitivity improvement, as shown in Figure 8b. The proposed sensor shows the improvement in the sensitivity with the values of 19.15% (1.12 times) and 9.8% (1.1 times) in comparison to references [40,52], respectively, when detecting the dielectric slab of 10. In light of the results presented in Table 3, Figure 8a,b, it is evident that the proposed coupling mechanism improves the sensitivity and enhances the coupling simultaneously.

**Figure 9:**
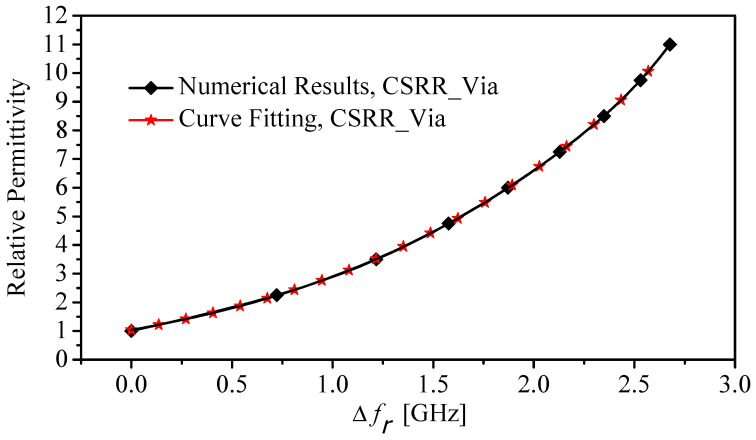
The relative permittivity versus the resonance frequency shift using the numerical simulation (HFSS) and the curve fitting.

By using fitting-function techniques, the relative permittivity of MUTs can be expressed in terms of the shifts in the resonance frequency as

(7) $$\epsilon_{r}=-0.462+38.139\times \left(\left(\frac{0.976}{1+e^{\left(\frac{\left(\Delta f-3.693\right)}{-1.1657}\right)}}\right)+\frac{\left(1-0.976\right)}{1+e^{\left(\frac{\left(\Delta f-2.667\right)}{-0.0995}\right)}}\right)$$

where Δf is the resonance frequency shift. Figure 9 presents two curves extracted using the numerical simulation (HFSS) and a fitting-function technique for the proposed sensor (CSRR with the via). Equation (7) can be utilized to characterize the real part of the dielectric constants of the MUTs. Note that (7) is only valid to characterize MUTs that have their dielectric constants between 1 and 11.

**Figure 10:**
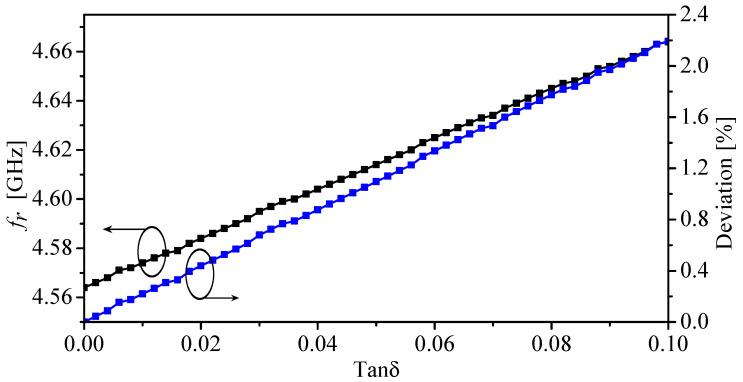
The resonance frequency and the deviation in the resonance frequency with respect to the resonance frequency in the case of the loss tangent of 0 versus the loss tangent, in the presence of $\epsilon_{r}$ = 2.

**Figure 11:**
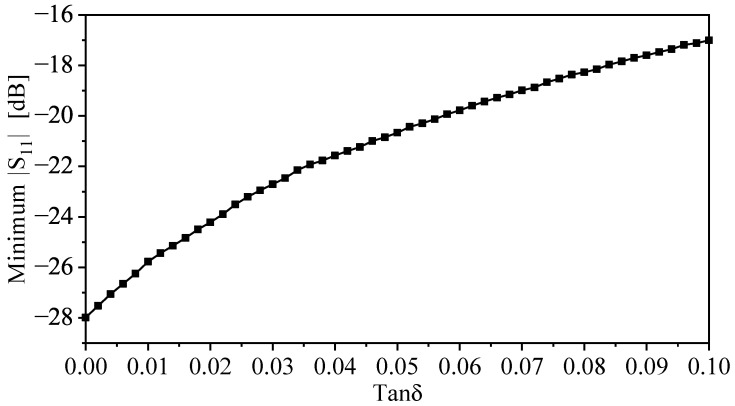
The minimum ($|S_{11}|$) versus the loss tangent, in the presence of $\epsilon_{r}$ = 2.

Furthermore, defected ground-based resonators such as CSRRs can be utilized to design near-field sensors. The sensing mechanism is based on observing resonance frequency shifts. Thus, by finding the relationship between the resonance frequency shifts and the real relative permittivity, a mathematical model can be constructed, and the sensors can be used for material characterization. However, the resonators can have some limitations if loaded with high-lossy materials (high-loss tangent) [40], where the resonance frequency will start to show a dependency on the loss tangent. This dependency will start to have a measurable effect on the resonance frequency. It is important, then, to determine the range of the loss tangent where the model can give acceptable deviations. Of course, it depends on the targeted applications and their tolerance towards certain deviations. It has been shown that for low-loss to moderately lossy materials, the deviation can increase up to 1% [40]. To study the effects of the losses in the materials on the resonance frequencies of the proposed sensor, the sensor was loaded with a dielectric slab with the dielectric constant of two and a loss tangent ranging from 0 to 0.1. Figure 10 shows the resonance frequency and the deviation versus the loss tangent. The deviation is calculated with respect to the resonance frequency in the case of the loss tangent of 0. From Figure 10, it can be observed that with the loss tangent of 0.09, the deviation is 2%. In addition, Figure 11 shows the minimum ($|S_{11}|$) versus the loss tangent.

## 4. Sensor Fabrication and Experimental Results

**Figure 12:**
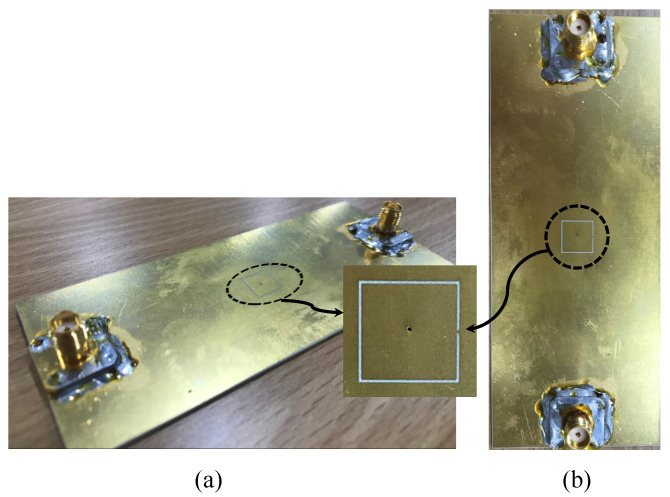
The fabricated CSRR sensor with a via. (**a**) Perspective view. (**b**) The top view.

**Figure 13:**
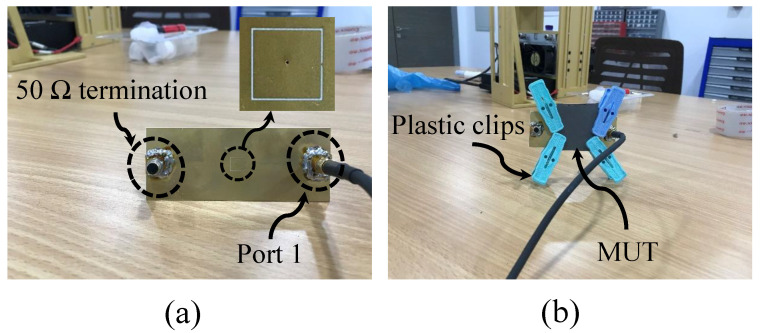
The experimental setup for measuring the response of the proposed sensor. (**a**) The proposed sensor terminated with a 50 Ω impedance, (**b**) where the sensor is placed in the free space.

For validation purposes, the proposed sensor (CSRR with a via) with the specification presented in Table 1 was fabricated using PCB technology. Figure 12 shows the top and perspective view of the sensor. The sensor was utilized to detect the presence of two dielectric slabs, Rogers RT/duroid 5870 and TMM10 laminates with dielectric constants of 2.3 and 9.2, respectively. The response of the sensor was measured using a VNA from Keysight (PNA-X network analyzer, N5242A, 10 MHz-26.5 GHz), shown in Figure 13a. The procedure started with terminating the sensor with 50 Ω impedance and placing it in free space, as shown in Figure 13b. To hold the slabs in position and to reduce the possible air gap between the slabs and the sensor, four plastic clips were used, as shown in Figure 13; however, the air gap cannot be completely avoided even with the utilization of techniques such as the ones that are presented in references [61,62], which can help to further reduce the effect of the air gap. However, errors in the measurement caused by the discrepancy between the model and the measurement must be calculated. Then, the VNA was utilized to record the response of the sensor ($|S_{11}|$) in the presence of free space, Rogers RT/duroid 5870, and TMM10 laminates, as shown in Figure 13a,b, respectively.

**Figure 14:**
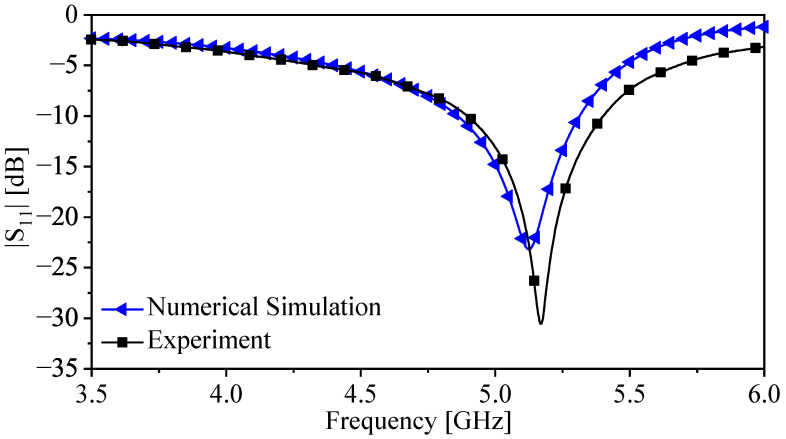
The response of the proposed sensor ($|S_{11}|$) extracted experimentally and using the numerical simulation (HFSS).

Figure 14 presents the response of the sensor extracted experimentally and using the numerical simulation in the presence of free space (the reference case). There is a relatively small deviation that can be associated with the fabrication tolerance. Since the detection mechanism is based on relative values, not absolute values such as $|S_{11}|$, the deviation can be tolerated and this is an advantage of using resonant structures-based sensors that are also less sensitive to the generated noise and undesired loss and phase shift during measurement [63]. Nevertheless, the error in the measurement must be calculated.

**Figure 15:**
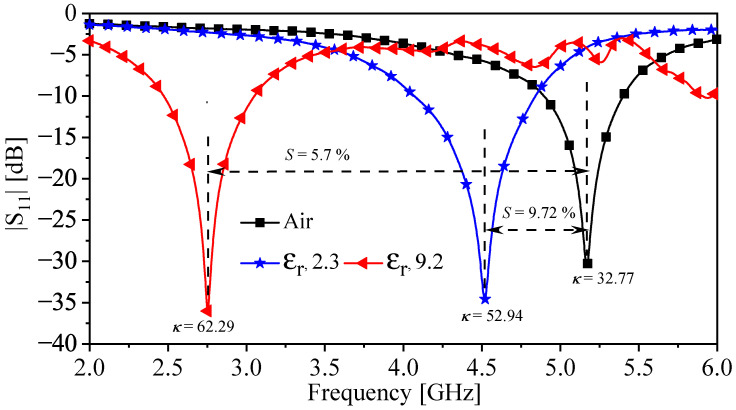
A descriptive response ($|S_{11}|$) extracted experimentally from the proposed sensor in the presence of the MUTs, the free space, Rogers RT/duroid 5870, and TMM10 laminates.

Figure 15 shows a descriptive response ($|S_{11}|$) extracted experimentally from the proposed sensor in the presence of the MUTs. From Figure 15, the resonance frequencies for the cases (free space, Rogers RT/duroid 5870, and TMM10 laminates) are 5.2 GHz, 4.52 GHz, and 2.75, respectively. By using (4)–(6), the calculated NormΔf, sensitivity (*S*), and the coupling factor (κ) are summarized in Table 4. Moreover, the sensor can be utilized to characterize the dielectric materials. This can be performed by substituting the calculated resonance shifts (extracted experimentally) in (7). The extracted values of the relative permittivity of the two slabs (2.3 and 9.2) are 2.15 and 9.17, respectively. Thus, the absolute errors in the extracted values are 6.52% and 0.33% for the two slabs (2.3 and 9.2), respectively. The error can be associated with the fabrication tolerance as well as the air gap between the sensor and the MUTs. The results are summarized in Table 4.

## 5. Conclusions

This paper presented a novel coupling mechanism for exciting a CSRR as a near-field dielectric sensor. The coupling is based on inductive coupling synthesized using a via that connects the power plane of a microstrip line to the central island of the resonator. The inductive coupling sensitizes the resonator’s capacitance to detect the changes in the MUTs compared to the coupling capacitance that is between the transmission line and the resonator. In addition, the coupling between the TL and the resonator is no longer dependent entirely on the capacitive coupling, which substantially reduces the coupling degradation caused by loading the resonator with MUTs. Thus, by using such a coupling mechanism, the sensitivity and the coupling factor were simultaneously improved. The proposed sensor was evaluated and quantified using the 3D simulation (HFSS) in which the normalized resonance shift, the sensitivity, and the coupling factor were analyzed. The proposed sensor shows a maximum sensitivity enhancement of 19.15%. The proposed coupling mechanism of the CSRR-based sensor was validated by fabricating the sensor using PCB technology and used to detect the two dielectric slabs, the Rogers RT/duroid 5870 and TMM10 laminates, with the dielectric constants of 2.3 and 9.2, respectively. In addition, the proposed sensor was utilized to characterize the real part of the dielectric constant of the two slabs (2.3 and 9.2) with absolute errors of 6.52% and 0.33%, respectively. The outcome of such results shows that the proposed sensor has the potential to be further investigated for future work and it is expected to be utilized in many sensing applications.

## Figures and Tables

**Table 1:** Design specification of the sensors.

Sensor Type	d (mm)	$W_{\mathrm{sub}}$ (mm)	$W_{\mathrm{TL}}$ (mm)	e (mm)	K (mm)	L (mm)	t (mm)	b (mm)
**CSRR (No Via)**	NA	0.762	1.629	40	100	7.5	0.2	0.2
**CSRR (with Via)**	0.2	∼	∼	∼	∼	∼	∼	∼

**Table 2:** Extracted lumped elements of the sensors.

Sensor Type	$L_{L}$ (nH)	$C_{L}$ (pF)	$R_{L}$ (kΩ)	$L_{\mathrm{via}}$ (nH)	$L_{r}$ (nH)	$C_{r}$ (pF)	$R_{r}$ (kΩ)
**CSRR (No Via)**	1.428	0.478	214.124	NA	3.248	0.272	5.783
**CSRR (with Via)**	1.942	105.727 × 10${}^{-6}$	2.056	0.678	1.380	1.138	1.774

**Table 3:** Table of comparison: The state-of-the-art sensors and the original CSRRs.

Ref.	$f_{r}$ [GHz]	Norm $\Delta f_{r}$ [%]	Sen. [%]	κ	Relative Size	Dims. (mm${}^{2}$)	Planar
[59] Rect. CSRR (7 mm)	2.325	$\Delta f_{r{|}_{\epsilon_{r}=2}}$ = 7.53 $\Delta f_{r{|}_{\epsilon_{r}=10}}$ = 34.95	$S_{{|}_{\epsilon_{r}=2}}$ = 7.53 $S_{{|}_{\epsilon_{r}=10}}$ = 3.88	NA	0.05 $\lambda_{0}$	7 × 7	Yes
[59] Circular CSRR (7 mm)	2.65	$\Delta f_{r{|}_{\epsilon_{r}=2}}$ = 6.6 $\Delta f_{r{|}_{\epsilon_{r}=10}}$ = 35.38	$S_{{|}_{\epsilon_{r}=2}}$ = 6.6 $S_{{|}_{\epsilon_{r}=10}}$ = 3.93	NA	0.05 $\lambda_{0}$	38.5	Yes
[60] IDC-SRR (7 mm)	2.45	$\Delta f_{r{|}_{\epsilon_{r}=2}}$ = 5.1 $\Delta f_{r{|}_{\epsilon_{r}=5}}$ = 17.34	$S_{{|}_{\epsilon_{r}=2}}$ = 5.1 $S_{{|}_{\epsilon_{r}=5}}$ = 4.335	NA	0.057 $\lambda_{0}$	7 × 4	Yes
[40] Optim. Shape (10.2 mm)	5.63	$\Delta f_{r{|}_{\epsilon_{r}=2}}$ = 10 $\Delta f_{r{|}_{\epsilon_{r}=10}}$ = 46	$S_{{|}_{\epsilon_{r}=2}}$ = 10 $S_{{|}_{\epsilon_{r}=10}}$ = 5.1	NA	0.2 $\lambda_{0}$	10.2 × 10.2	Yes
[52] Coupled 4CSRRs (7.5 mm)	4.317	$\Delta f_{r{|}_{\epsilon_{r}=2}}$ = 10 $\Delta f_{r{|}_{\epsilon_{r}=10}}$ = 42.27	$S_{{|}_{\epsilon_{r}=2}}$ = 10 $S_{{|}_{\epsilon_{r}=10}}$ = 4.7	NA	0.11 $\lambda_{0}$	31.5 × 7.5	Yes
Original CSRR (No Via) (7.5 mm)	3.22	$\Delta f_{r{|}_{\epsilon_{r}=2}}$ = 8.06 $\Delta f_{r{|}_{\epsilon_{r}=10}}$ = 37.74	$S_{{|}_{\epsilon_{r}=2}}$ = 8.06 $S_{{|}_{\epsilon_{r}=10}}$ = 4.2	$\kappa_{{|}_{\epsilon_{r}=2}}$ = 14.86 $\kappa_{{|}_{\epsilon_{r}=10}}$ = 6.25 $\kappa_{{|}_{\epsilon_{r}=19}}$ = 3.15 $\kappa_{{|}_{\epsilon_{r}=30}}$ = 1.55 $\kappa_{{|}_{\epsilon_{r}=69}}$ = 0.42 $\kappa_{{|}_{\epsilon_{r}=80}}$ = 0.35	0.08 $\lambda_{0}$	7.5 × 7.5	Yes
Original CSRR (No Via) (10.2 mm)	2.366	$\Delta f_{r{|}_{\epsilon_{r}=2}}$ = 8 $\Delta f_{r{|}_{\epsilon_{r}=10}}$ = 37.36	$S_{{|}_{\epsilon_{r}=2}}$ = 8 $S_{{|}_{\epsilon_{r}=10}}$ = 4.15	$\kappa_{{|}_{\epsilon_{r}=2}}$ = 17.12 $\kappa_{{|}_{\epsilon_{r}=10}}$ = 6.733 $\kappa_{{|}_{\epsilon_{r}=19}}$ = 2.93 $\kappa_{{|}_{\epsilon_{r}=30}}$ = 1.6 $\kappa_{{|}_{\epsilon_{r}=69}}$ = 0.46 $\kappa_{{|}_{\epsilon_{r}=80}}$ = 0.35	0.08 $\lambda_{0}$	10.2 × 10.2	Yes
**CSRR (Via) ** **(7.5 mm) (T.W.)**	**5.092**	**$\Delta f_{r{|}_{\epsilon_{r}=2}}$ = 12** **$\Delta f_{r{|}_{\epsilon_{r}=10}}$ = 50.34**	**$S_{{|}_{\epsilon_{r}=2}}$ = 12** **$S_{{|}_{\epsilon_{r}=10}}$ = 5.6**	**$\kappa_{{|}_{\epsilon_{r}=2}}$ = 22.95** **$\kappa_{{|}_{\epsilon_{r}=10}}$ = 92.05** **$\kappa_{{|}_{\epsilon_{r}=19}}$ = 88.1** **$\kappa_{{|}_{\epsilon_{r}=30}}$ = 73.55** **$\kappa_{{|}_{\epsilon_{r}=69}}$ = 39** **$\kappa_{{|}_{\epsilon_{r}=80}}$ = 36.2**	**0.127 $\lambda_{0}$**	**7.5** × **7.5**	**Yes**
**CSRR (Via)** ** (10.2 mm) (T.W.)**	**3.763**	**$\Delta f_{r{|}_{\epsilon_{r}=2}}$ = 10.8** **$\Delta f_{r{|}_{\epsilon_{r}=10}}$ = 49.5**	**$S_{{|}_{\epsilon_{r}=2}}$ = 10.8** **$S_{{|}_{\epsilon_{r}=10}}$ = 5.5**	**$\kappa_{{|}_{\epsilon_{r}=2}}$ = 21.88** **$\kappa_{{|}_{\epsilon_{r}=10}}$ = 108.4** **$\kappa_{{|}_{\epsilon_{r}=19}}$ = 86.45** **$\kappa_{{|}_{\epsilon_{r}=30}}$ = 59.8** **$\kappa_{{|}_{\epsilon_{r}=69}}$ = 34.8** **$\kappa_{{|}_{\epsilon_{r}=80}}$ = 30.26**	**0.127 $\lambda_{0}$**	**10.2** × **10.2**	**Yes**

**Table 4:** The summarized results based on the experiment.

MUT	Norm Δf [%]	Sensitivity (*S*) [%]	Coupling Factor (κ)	The Extracted $\epsilon_{r}$	The Absolute Errors [%]
($\epsilon_{r}=2.3$)	12.63	9.72	52.94	2.15	6.52
($\epsilon_{r}=9.2$)	46.74	5.7	62.29	9.17	0.33

## Data Availability

Data generated during the study are contained within the article.
